# Decitabine Induced Delayed Cardiomyopathy in Hematologic Malignancy

**DOI:** 10.1155/2018/3953579

**Published:** 2018-09-30

**Authors:** Pradyumna Agasthi, Hemalatha Narayanasamy, Dan Sorajja, James Slack, Farouk Mookadam

**Affiliations:** ^1^Department of Cardiovascular Diseases, Mayo Clinic, Arizona 85259, USA; ^2^Department of Cardio-Oncology, Mayo Clinic, Arizona 85259, USA; ^3^Department of Internal Medicine, Mayo Clinic, Arizona 85259, USA

## Abstract

Decitabine is a pyrimidine analogue of nucleoside cytidine, used for the treatment of myelodysplastic syndromes, chronic myelogenous leukemia, and acute myelogenous leukemia. We present a case of cardiomyopathy associated with decitabine used for secondary acute myelogenous leukemia. The patient presented with new heart failure symptoms and an ejection fraction decline.

## 1. Introduction

Decitabine is a pyrimidine analogue of the nucleoside cytidine [1]. It inhibits DNA methylation and plays a role in reversing epigenetic silencing in malignant cells. Decitabine is currently indicated in the treatment of myelodysplastic syndromes (MDS) [2–4]. To our knowledge, one case of myocarditis [5] and one case of reversible nonischemic cardiomyopathy [6] from decitabine have been reported, this being the 3rd case.

## 2. Case Report

A 71-year-old female with a history significant for hypertension, hepatitis B, and hypothyroidism, underwent bone marrow biopsy which showed a hypercellular bone marrow with >90% cellularity and 81% myeloblasts expressing CD 34 and CD 117 markers, confirming a diagnosis of acute myeloid leukemia (AML). Molecular testing showed no evidence for FMS-like tyrosine kinase 3 internal tandem duplication, absence of nucleophosmin1 and KIT exon 8, and 17 mutations, suggesting a lower risk of relapse after chemotherapy. Based on cytogenetic studies, secondary AML was diagnosed. Given her advanced age, decitabine therapy was commenced. A baseline 2-D transthoracic echocardiogram (TTE) showed normal function with an ejection fraction (EF) of 55–60%. After completing 10 cycles of decitabine, she was noted to have a tachycardia and dyspnea by self-report. She was therefore referred to cardiology with these symptoms in preparation for allogeneic stem cell transplant.

The heart rate was 110/min, and a 2/6 ejection systolic murmur and a loud P2 with an S3 and S4 gallop were heard. Lungs were clear. No jugular venous distension or pedal edema was noted.

**Figure 1:**
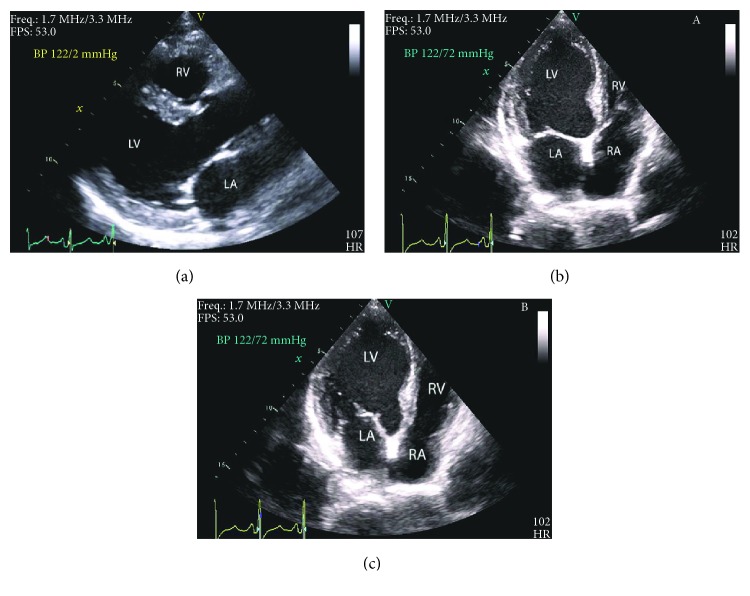
Transthoracic echocardiogram of the patient in parasternal long axis view (a) demonstrating an enlarged left ventricular cavity, in apical (four-chamber) view demonstrating a mildly abnormal end systolic (b) and normal end diastolic (c) left ventricular dimension (RV: right ventricle; LV: left ventricle; LA: left atrium; RA: right atrium).

**Figure 2:**
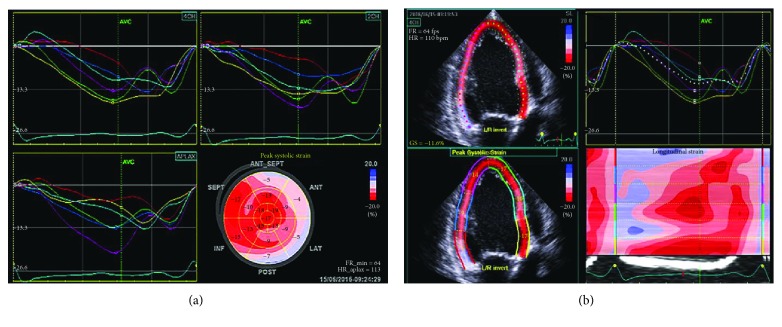
Peak systolic strain pattern in cross-sectional view (a) and longitudinal view (b) demonstrates decrease in left ventricular strain pattern.

Laboratory data is significant for a serum creatinine level of 0.8 mg/dl, estimated glomerular filtration rate of 80 ml/min per 1.73 m^2^, and N-terminal pro-b-type natriuretic peptide level of 517 pg/ml. Her complete blood count showed a white blood cell count of 12.6 × 10^9^/l with greater than 50% blasts, low hemoglobin at 7.8 g/dl, hematocrit value of 25%, large platelet count of 212 × 10^9^/l, and lactate dehydrogenase level of 588 U/l. Serum troponin or creatinine phosphokinase levels were not performed due to a lack of discernibility in patients undergoing chemotherapy for cancer. Echocardiogram showed severe left ventricular systolic dysfunction (EF 28%), mildly abnormal end systolic dimension (Figure 1), and a mild reduction in right ventricular systolic function. Global averaged left ventricular longitudinal peak systolic strain was abnormal at −12% (normal more negative than −18%) (Figure 2). Nuclear stress test showed no evidence of coronary disease. Patient was euthyroid at the time of diagnosis.

The patient was diagnosed with New York Heart Association class II and American Heart Association stage B heart failure with reduced ejection fraction. In the absence of any viral illness, toxins, or coronary disease, or concomitant cardiotoxic medication use and known recent normal ejection fraction, the etiology was attributed to decitabine use. The patient was subsequently started on metoprolol succinate 50 mg twice a day and furosemide 20 mg daily. Follow-up echocardiogram 4 weeks later showed no change in the ejection fraction of 28%, but there was mild improvement in the global left ventricular longitudinal peak systolic strain at −15% (improved from −12%).

## 3. Discussion

### 3.1. Decitabine

Decitabine is an azanucleoside (AZN), a pyrimidine analogue of cytidine. Cancer cells often exhibit severely altered chromatin structure and methylation patterns leading to promoter hypermethylation and silencing of key tumor suppressor genes. The inactivation of tumor suppressor genes and other critical genes leads to unopposed proliferation of cancer cells [7–9].

Low dose AZNs have shown optimal inhibition of DNA methylation leading to subsequent cellular differentiation [10]. Decitabine inhibits the action of DNA methyltransferase and restores the normal growth and differentiation of cells [11]. Decitabine was approved in 2006 for high-risk myelodysplastic syndromes of all French-American-British subtypes, chronic myelogenous leukemia, acute myelogenous leukemia, and sickle cell anemia [3, 6, 12]. The common cardiovascular adverse effects ascribed to decitabine include peripheral edema (25%), cardiac murmurs (16%), chest discomfort (7%), and hypotension (6%) [4, 6].

Reports of decitabine-related cardiotoxicity in the literature are scant with a case of myocarditis in a 50-year-old female treated with azacitidine and decitabine for MDS [5]. A reversible nonischemic cardiomyopathy from decitabine in a 75-year-old patient treated with decitabine for AML, presented with heart failure and an EF of 35%. Left heart catheterization showed no obstructive coronary artery disease. With guideline-based heart failure therapy, EF normalised [6].

We present a 71-year-old female treated with AML for 10 cycles presenting with symptoms of EF decline.  Table 1 [6] shows the comparison of the previously reported reversible cardiomyopathy.

Approval for the use of decitabine in the treatment of myelodysplastic syndromes are based on two clinical trials [3, 13]. The number of patients in the clinical trials were 170 and 66, respectively. Mean ages of the patients were 68 yrs and 70 yrs. None of the patients participating in the clinical trials developed cardiomyopathy or heart failure. However, no routine echocardiograms or biomarkers were used during the study. The literature suggests that the mechanism for cardiomyopathy is likely myocarditis based on patent coronaries on coronary angiogram and biomarker elevation [6]. The current package insert mentions cardiomyopathy as a potential side effect without the suggestion that it may be attributable to myocarditis [14]. A recent retrospective study of 111 patients with AML treated with decitabine showed a higher incidence of cardiotoxic side effects in patients with renal dysfunction. New onset heart failure was noted only in patients with renal dysfunction [15]. Our patient developed cardiac dysfunction despite normal kidney function. Contrary to prior reports, our patient had a delayed onset of cardiac dysfunction, which is unique. Clearly, the etiology for cardiac dysfunction with decitabine therapy may be bimodal (early onset versus delayed) and may be due either to hypersensitivity to hypomethylating agents causing myocarditis or to cardiac cell injury. This has yet to be elucidated. We hypothesize that the reason for the lack of cardiac function recovery in our patient might be directly related to the extent or duration of cardiomyocyte injury endured over the prolonged course of decitabine therapy.

The prevalence of chemotherapy-induced cardiomyopathy is increasing because of longer cancer survivorship and multiple new chemotherapeutic regimens where cardiac side effects are understudied. Early detection and prompt treatment is the best strategy. Many of the chemotherapeutic agents have not been studied a priori for cardiotoxicity and are symptom driven. Cardiac dysfunction symptoms may mimic the side effects attributable to the chemotherapy and no standard screening guidelines exist, especially for newer drugs.

Lessons learned from the monoclonal antibody, trastuzumab, which was shown to have a much higher incidence of cardiotoxicity post marketing, need to be applied to newer agents. Well-designed clinical trials involving chemotherapeutic agents or registries for chemotherapeutic agents should be studied for the potential for cardiotoxicity.

## 4. Conclusion

We present a third case of cardiomyopathy with decitabine used for secondary AML. Since older populations are more prone to chemotherapy-associated cardiomyopathy, screening these vulnerable groups may be helpful for early diagnosis and early treatment.

## Figures and Tables

**Table 1:** Baseline characteristics of patients.

	Age/sex	Comorbid conditions	Decitabine	EF 1^a^	Biomarker	Test^b^	EF 2^c^	EF Δ^d^
De et al. [6] (2012)	75 yrs/M	DM type II, DVT	2 cycles	35%	BNP: 259	Coronary angiogram	50% (after 7 days)	Yes
Our patient	71 yrs/F	HTN, Hep B, hypothyroidism	10 cycles	28%	Pro-BNP: 517	Nuclear stress test	28% (after 4 weeks) but with improvement in strain	No

^a^Baseline left ventricular ejection fraction. ^b^Test to rule out coronary artery disease. ^c^Repeat left ventricular ejection fraction. ^d^Reversibility of ejection fraction. Note: both patients received decitabine as part of treatment for acute myeloid leukemia. Abbreviations: BNP: brain natriuretic peptide; DM: diabetes mellitus; DVT: deep vein thrombosis; EF: ejection fraction; HTN: hypertension; Hep B: hepatitis B; BNP units: pg/ml.
